# Impact of Teleconsultation on Patients With Type 2 Diabetes in the Brazilian Public Health System: Protocol for a Randomized Controlled Trial (TELEconsulta Diabetes Trial)

**DOI:** 10.2196/23679

**Published:** 2021-01-21

**Authors:** Daniela Laranja Gomes Rodrigues, Gisele Silvestre Belber, Frederica Valle De Queiroz Padilha, Ligia Fonseca Spinel, Frederico Rafael Moreira, Marcos Aurélio Maeyama, Ana Paula Neves Marques Pinho, Álvaro Avezum Júnior

**Affiliations:** 1 Hospital Alemão Oswaldo Cruz São Paulo Brazil; 2 Social Responsability Centre Hospital Alemáo Oswaldo Cruz São Paulo Brazil; 3 Innovation, Research and Education Institute Hospital Alemão Oswaldo Cruz São Paulo Brazil; 4 Telehealth Center Santa Catarina Brazil

**Keywords:** remote consultation, diabetes mellitus, telemedicine, telehealth, costs, public health

## Abstract

**Background:**

Although the Brazilian Unified Health System (SUS) offers universal health coverage, access to quality care is often limited by social inequality and location. Although telemedicine has been shown to be an important tool in the efforts to overcome this problem, because it can provide access to specialist care and break the geographical barriers to health care, there are no national studies demonstrating its use in public health.

**Objective:**

This study aims to test the hypothesis that remote consultation can be as effective as standard face-to-face consultation for type 2 diabetes mellitus in the Brazilian public health system and to assess the associated costs related to teleconsultation in public health scenarios, for patients referred from Primary Health Care units of the SUS for specialist care.

**Methods:**

This is a pragmatic, phase 2, unicentric, open-label, noninferiority, blinded allocation, data-blinded, centrally randomized clinical trial. The inclusion criteria will be adults, both sexes, ≥18 years old, glycated hemoglobin (HbA_1c_) ≥8%. Outcomes will be evaluated by assessing symptoms, laboratory exams, anthropometric measurements, blood pressure, adverse events, and satisfaction level for 6 months. The costs of the teleconsultation will be assessed using the time-driven activity-based costing (TDABC) method to compare the costs with the face-to-face consultations. The noninferiority margin was set at 0.5%. Assuming an SD of 1.3% for both groups, true difference between the means of zero, and a type I error level of 5% (one-sided), it was estimated that 117 individuals per group would be necessary to achieve 90% power. Statistical analysis of the efficacy will be done using intention-to-treat and per-protocol approaches.

**Results:**

The results from this trial will be reported according to the CONSORT guidelines. The trial was approved by the institutional review board on October 5, 2019. Data collection started in January 2019 and is expected to finish in 2022. At the time of manuscript submission, 18 participants were recruited.

**Conclusions:**

Our expectations are that providing remote access to health care will result in improvements in the health and quality of life of patients with type 2 diabetes and reduce costs and that both patients and clinicians will benefit from and be satisfied with this technology.

**Trial Registration:**

Registro Brasileiro de Ensaios Clínicos RBR-8gpgyd; https://ensaiosclinicos.gov.br/rg/RBR-8gpgyd

**International Registered Report Identifier (IRRID):**

DERR1-10.2196/23679

## Introduction

According to the 2016 Global Burden of Disease [1], despite the increase in both quality of life and access to health care observed since 1990, there are still many countries in which health inequalities remain, particularly in relation to cancer and noncommunicable diseases, such as asthma, chronic obstructive pulmonary disease, and diabetes. These diseases have a significant impact on quality of life [2], and this is particularly true in the case of diabetes, because it has a range of serious complications in situations where it is poorly controlled by the individual, such as cardiovascular disease, chronic kidney disease, blindness, and lower limb amputation [3], many of which could be avoided by providing better access to health care.

**Figure 1:**
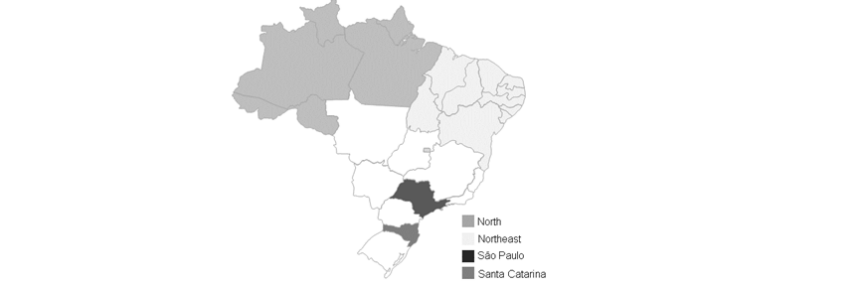
Regions of Brazil related to this project.

In 2016, Brazil was ranked 96th among 195 countries regarding access to health care and quality of life, according to the Global Burden of Disease [1]. Despite a slight improvement in the health index (from 46.5 in 1990 to 63.8 in 2016), access to health care is still one of the largest indicators of social inequality [4]. While the inhabitants of São Paulo, Brazil’s largest city (Figure 1), have access to 2.81 doctors per 1000 inhabitants, in the northeast region, this proportion is about 1.41 doctors, and in some states, this proportion can even reach <1 doctor per 1000 inhabitants [4]. When considering physicians of all medical specialties, almost 70% of these professionals are concentrated in the south and southeast regions of the country [5]. To improve medical access, there is a need to develop strategies that can not only enhance local primary care but also improve the regulatory processes and the organization of specialized health care in Brazil [6-8].

Although there is increasing evidence for the use of remote consultation worldwide [9-11], in Brazil, under Resolution number 1643/2002 [12] of the Federal Council of Medicine, medical consultations by telephone or internet cannot be conducted directly between health professionals and patients. Thus, currently, teleconsulting is only allowed when there is a health professional at both ends of the communication channel, except in the COVID-19 pandemic situation. Also, there are no national studies in Brazil that address the effectiveness and costs involved in teleconsultation service in the public health system. If teleconsulting directly between the physician and the patient can be shown to be safe and effective, this would provide strong evidence to change the current regulations, freeing up one professional from the consultation and driving down costs. The main objective of this study is to test the hypothesis that remote consultation can be as effective as standard face-to-face consultation for type 2 diabetes in the Brazilian public health system and to assess the associated costs related to teleconsultation in public health scenarios.

## Methods

TELEConsult Diabetes is a pragmatic, phase 2, single-center, open-label, noninferiority trial with central randomization that will evaluate the efficacy and safety of specialized remote consultation compared to face-to-face consultation in patients with type 2 diabetes mellitus referred by primary health care units to specialist care in the Unified Health System (SUS) [13].

The inclusion criteria are adults of both sexes, ≥18 years old, established diagnosis of type 2 diabetes, and either not insulin dependent (with any level of glycemic control measured by glycated hemoglobin [HbA_1c_] accordingly to local protocol [14-17]) or insulin dependent (HbA_1c_ >8%). Exclusion criteria are patients <18 years old or with type 1 diabetes, women with gestational diabetes or diagnosed during pregnancy, and patients with chronic renal failure with estimated or measured creatinine clearance <30 mL/min/m^2^ [18].

Brazil has a universal public health care system called SUS, which is structured around the principle of primary care and operates by integrating other services and levels of care, such as specialized care, into the system for the whole population. The main goal of primary care in Brazil is to provide basic care and to coordinate other levels of care throughout the health system network, regulating referrals to specialist care [8].

The Telehealth Center of Santa Catarina state uses a system that does not represent the reality in most Brazilian states. First, the primary care physicians request a teleconsultation with a specialist. This consultation involves both the primary care physicians and the specialist. Following this first contact, the primary care physician decides whether the patient needs to be referred to a face-to-face consultation with the specialist [19,20]. This system of compulsory flow established and operating in Santa Catarina state is the ideal setting to investigate the potential of remote consultation in primary health care, compared to face-to-face consultations.

The participant will be identified by the primary care physician and the Telehealth Center of the state of Santa Catarina, and the regulatory department of the Municipal Health Office of Joinville will determine if she or he meets the eligibility criteria. Patients with type 2 diabetes mellitus may be recruited from any of the city’s 70 primary care centers. A researcher will contact the patient by telephone to tell them about the trial and invite them to participate in the study. If the patient agrees, they will be told that they need to provide their written consent, and an informed consent form will be posted to them prior to reading and assessment for their participation in the study. Then, 2-5 days after sending the informed consent form, the researcher will again telephone the patient to confirm their interest in participating in the trial. If the response is still positive, they will schedule a home visit at a convenient time with the patient, in the presence of a legal representative or relative if they so wish. During the visit, any questions related to the study will be answered. After signing and agreeing to participate in this study, the researcher will tell the participant about the next steps: Their data will be randomized in a system created especially for this trial, and they will be scheduled for a face-to-face consultation or for a remote consultation with the specialist. By the end of this home visit, the participant will have been advised about the study, their participation, the date and place of the research consultation, and that there are no costs involved.

To calculate the real cost of a teleconsultation service to compare with the cost of the face-to-face service and to generate a cost parameter for the primary health care units for this type of service, the time-driven activity-based costing (TDABC) method will be used [21,22]. In addition to the costs of the resources used, this method also considers the amount of time spent on each stage of the process, enabling the identification of stages where the teleconsultation speeds up or slows down the process, reducing or increasing costs in relation to face-to-face consultation.

This study will also include other indicators, such as the transportation costs for the patients and physicians in a “real-word” scenario, to carry out cost-effectiveness estimates. Finally, we intend to discuss issues related to the reduced costs that increased access to medical care, via teleconsultation, can generate for the system.

The randomization list will be generated electronically using appropriate software. Randomization will be performed in blocks of 4 at a 1:1 ratio. Confidentiality of the randomization list will be ensured by setting up a central database and the use of an electronic case report form. Access to the system will be granted through specific usernames and passwords given to each investigator or study team. The patient will be allocated to one of the treatments (remote consultation or face-to-face consultation) only after being registered in the system. Given the nature of the intervention, blinding is not feasible. However, data analysis will be performed by a statistician blind to patient allocations.

Participants in both groups will be assisted by 1 of a team of 4 endocrinologists from the public outpatient clinic. They will take turns to provide assistance in the 2 modalities. The remote consultation with the intervention group will involve only the specialist and the participant. These consultations will take place in 1 of the 6 primary care units chosen to cover the main regions of the city. In each of these units, the research team set up a room with a computer, microphone, and camera to deliver the remote consultation, assisted by the same team of endocrinologists.

The same physicians will undertake both the remote consultation and the face-to-face consultation, with the same duration (30-60 minutes). All the physicians are specialists medically trained in endocrinology and experienced in caring for patients with diabetes in different settings and as part of the city’s specialist care network. A protocol based on national and international guidelines specifically designed for the study [17,23-25] will be used to ensure that all the consultations are as similar as possible. The physicians were instructed to follow the protocol to avoid any bias related to the consultation. The protocol includes instruction on the questions to be asked about the participant’s health and the medicines they are using, the scale to be used for hypoglycemia evaluation [26], and the medications to be prescribed (following current guidelines). At the end of the consultation, participants from both groups receive, in addition to medical advice and drug prescriptions, guidance on any laboratory tests that need to be carried out before the next consultation.

The primary outcome will be a change in HbA_1c_ levels at 6 months after randomization. However, we will perform a prespecified analysis 3 months after randomization.

Secondary outcomes will be fasting blood glucose, complete blood count, urea, creatinine, total cholesterol, triglycerides, high-density lipoprotein cholesterol, low-density lipoprotein cholesterol, systolic and diastolic blood pressure, body weight, BMI, frequency of hypoglycemia, and incidence of adverse events. In addition, in the intervention group, we intend to evaluate the satisfaction of the endocrinologists and the patients with the video conference system using a structured questionnaire [23].

A specific quality of life in diabetes questionnaire will be used with patients with diabetes mellitus in the first and last research consultations in both groups [27-29]. In addition, we will estimate the actual cost of the remote consultation service, using the TDABC method [21,22] to obtain the unit cost of remote consultation in the primary health care units.

This trial is registered with the Ethics and Research Commission (03434218.1.2001.5362).

## Results

This study was approved and funded by the Brazilian Ministry of Health in October 2018 and was approved by the institutional review board in October 2019. Data collection started in January 2019 and is expected to finish in 2022. At the time of manuscript submission, 18 participants were recruited.

The primary objective of this study is to confirm the noninferiority of remote consultation in comparison with face-to-face consultation assessed by the change from baseline in HbA_1c_ (%) at 6 months. The noninferiority margin was set at 0.5%. Assuming an SD of 1.3% for both groups, true difference between the means of zero, and a type I error level of 5% (one-sided), it was estimated that 117 individuals per group would be necessary to achieve 90% power [30]. In order to accommodate for a maximum dropout rate of 5%, the sample size was increased to 124 individuals per group. Sample size calculation was performed using SAS version 9.4 (SAS Institute Inc, Cary, NC).

The primary endpoint in this study is the change from baseline in HbA_1c_ (%) at 6 months. The assessment of noninferiority of remote consultation in relation to face-to-face will be conducted according to the CONSORT guidelines [31], using the confidence interval approach to the difference in the mean of the primary variable between the 2 groups. If the upper limit of the 90% bilateral CI is lower than the established noninferiority margin (0.5%), the noninferiority of the remote consultation group relative to the face-to-face group is declared at the 5% significance level. In addition, an analysis of covariance (ANCOVA) model will be constructed using the main effects of treatment and baseline HbA_1c_ as the covariate.

Adjusted means by treatment will be presented as well as an estimate of the difference between adjusted means. A 90% 2-sided CI, based on the ANCOVA model, will be computed for the difference between adjusted means. If the upper limit of the 90% bilateral CI is lower than the established noninferiority margin (0.5%), the noninferiority of the remote consultation group relative to the face-to-face group is declared at the 5% significance level [32-34]. The primary endpoint will be analyzed for the intention-to-treat (ITT) and per-protocol populations. If the proportion of missing data is greater than 5%, sensitivity analyses for missing data imputation will be performed [35,36].

The secondary endpoint is the change from baseline in HbA_1c_ at 3 months, which will be evaluated with ANCOVA. Adjusted means (with 95% CI) by treatment will be presented. Mixed effects, repeated measures models will be considered for secondary endpoints defined by continuous variables over time (baseline, 3 months, 6 months). Comparison of secondary endpoints defined by categorical data will be evaluated using chi-square or Fisher exact tests. Secondary endpoints will be performed as 2-sided tests with an alpha of 5%, and 95% CIs will be reported. The proportion of adverse events will be compared between the 2 groups via a Fisher exact test. The secondary endpoints will be analyzed on the ITT population.

Baseline characteristics will be compared and summarized by treatment groups for the ITT population. Categorical data will be summarized by numbers and percentages. Continuous data will be summarized by mean, SD, and range if data are normal and median and IQR if data are skewed. Normality will be assessed by visual inspection of histograms and with the Shapiro-Wilk normality test [36]. Baseline variables will be compared with the chi-square test or Fisher exact test for categorical variables and the *t* test or Mann-Whitney test for continuous variables. Statistical analyses will be performed using SAS version 9.4.

## Discussion

Brazil is a country of continental proportions, with great heterogeneities and gaps in access to health care, types of health services, and specialized medical professionals. Lack of access to health services is one of the main indicators of social inequality in Brazil. Thus, to improve access, there is a need to build strategies that impact primary health care, the processes that regulate access, and the organization of specialist care. In this context, studies have shown telemedicine to be equivalent to face-to-face care, and it can be an effective solution to increasing patient access to services, especially to specialist doctors.

Providing evidence of the efficacy and safety of remote treatment for different conditions in Brazil will contribute to improving patients’ access to the public health system, including specialist doctors. This evidence can also help to remove restrictions placed on remote consultation by the Brazilian Federal Medicine Council, which currently restricts direct specialist-to-patient consultation. This model of improved access can help to meet the health needs of the population, breaking the geographical barriers that a country like Brazil imposes on the provision of health services. In addition to greater access to health care, the use of telemedicine has potential economic benefits for health systems and can be used safely to deliver a quality service.

Our expectations are that providing remote access to health care will result in improvements in the health and quality of life of patients with type 2 diabetes and reduce costs and that both patients and clinicians will benefit from and be satisfied with this technology.

## Abbreviations

ANCOVA: analysis of covariance
HbA_1c_: glycated hemoglobin
ITT: intention-to-treat
SUS: Unified Health System (in Portuguese)
TDABC: time-driven activity-based costing
